# Real‐time fluorometric evaluation of hepatoblast proliferation in vivo and in vitro using the expression of *CYP3A7* coding for human fetus‐specific P450

**DOI:** 10.1002/prp2.642

**Published:** 2020-09-04

**Authors:** Shota Okuyama, Fumihiko Kawamura, Musashi Kubiura, Saori Tsuji, Mitsuhiko Osaki, Hiroyuki Kugoh, Mitsuo Oshimura, Yasuhiro Kazuki, Masako Tada

**Affiliations:** ^1^ Stem Cells & Reprogramming Laboratory Department of Biology Faculty of Science Toho University Funabashi Japan; ^2^ Institute of Regenerative Medicine and Biofunction Graduate School of Medical Science Tottori University Yonago Japan; ^3^ Chromosome Engineering Research Center Tottori University Yonago Japan

**Keywords:** hepatoblasts, hepatocytes, human CYP3A7, mouse embryonic stem cells, reporter genes, transgenic mice

## Abstract

The fields of drug discovery and regenerative medicine require large numbers of adult human primary hepatocytes. For this purpose, it is desirable to use hepatocyte‐like cells (HLCs) differentiated from human pluripotent stem cells (PSCs). Premature hepatoblast‐like cells (HB‐LCs) differentiated from PSCs provide an intermediate source and steady supply of newly mature HLCs. To develop an efficient HB‐LC induction method, we constructed a red fluorescent reporter, CYP3A7R, in which *DsRed* is placed under the transcriptional control of *CYP3A7* coding for a human fetus‐type P450 enzyme. Before using this reporter in human cells, we created transgenic mice using mouse embryonic stem cells (ESCs) carrying a CYP3A7R transgene and confirmed that CYP3A7R was specifically expressed in fetal and newborn livers and reactivated in the adult liver in response to hepatic regeneration. Moreover, we optimized the induction procedure of HB‐LCs from transgenic mouse ESCs using semi‐quantitative fluorometric evaluation. Activation of Wnt signaling together with chromatin modulation prior to Activin A treatment greatly improved the induction efficiency of HB‐LCs. BMP2 and 1.7% dimethyl sulfoxide induced selective proliferation of HB‐LCs, which matured to HLCs. Therefore, CYP3A7R will provide a fluorometric evaluation system for high content screening of chemicals that induce HB‐LC differentiation, hepatocyte regeneration, and hepatotoxicity when it is introduced into human PSCs.

**Abbreviations**
EMT: epithelial‐mesenchymal transition
ESCs: embryonic stem cells
HB‐LCs: hepatoblast‐like cells
HLCs: hepatocyte‐like cells
iPSCs: induced pluripotent stem cells

## 1 INTRODUCTION

Drugs are primarily metabolized in the liver, and in humans, 50 to 60% of the oxidation of clinical drugs is achieved by CYP3A4, the major P450 enzyme. However, expression of *CYP3A4* is spatially and developmentally restricted to hepatocytes located around the central region of the hepatic lobule Zone 3 in adults.^1^ Therefore, there is a demand for CYP3A4‐expressing (CYP3A4^+^) HLCs to use as model cells in place of primary human hepatocytes for preclinical studies to eliminate cytotoxic and ineffective drugs. Human ESCs and induced PSCs (iPSCs) provide a limitless supply of HLCs through hepatic cell differentiation. The primary requirement for primary human hepatocyte replacement is the full expression of CYP3A4, but the metabolic activity of PSC‐derived HLCs is much lower than that of in vivo hepatocytes.^2^, ^3^ Recently, three‐dimensional (3D) cell cultures have been used to provide higher metabolic activity to immature hepatocytes, but the resulting cells are still far inferior to primary human hepatocytes.^4^, ^5^, ^6^

Hepatoblasts are parenchymal cells of fetal livers and are capable of self‐renewal in culture and differentiation into hepatocytes or biliary epithelium. Therefore, large numbers of HB‐LCs can be stored as an intermediate source of CYP3A4^+^ HLCs. HB‐LCs will also be of great help in improving the HLC maturation method. To allow the efficient production of functional HB‐LCs, we sought to develop a reporter system for the identification of cells possessing similar characteristics to those of in‐vivo fetal hepatic parenchymal cells.

CYP3A7, a major fetal‐type P450 enzyme is mainly expressed in the human fetal liver and intestine and replaces the CYP3A4 function.^7^ In addition, CYP3A7 is expressed in placenta, and occasionally contributes to the teratogenicity of embryos and birth defects, such as when teratogens like thalidomide are administered. The human *CYP3A7* transgene can reproduce the tissue‐specific and developmentally regulated expression in mice.^8^ We previously constructed a BAC vector to express RFP under the *CYP3A7* promoter (CYP3A7R) and GFP under the *CYP3A4* promoter (CYP3A4G), and were able to produce transgenic HepaRG cells. HepaRG cells are bipotent HB‐like hepatocellular carcinoma cells capable of differentiating into HLCs and biliary epithelium after 2 weeks of culturing in the presence of 1.7‐2.0% dimethyl sulfoxide (DMSO).^9^, ^10^ A majority of transgenic HepaRG cells express CYP3A7 and RFP in the HB‐LC state before developing into CYP3A4^+^ and GFP^+^ HLCs. Therefore, in the current study, we first used mice to determine whether expression of CYP3A7R can be used to report accurately hepatoblasts in vivo and HB‐LCs in vitro.

During embryonic development, hepatoblasts originate from the foregut definitive endoderm and form the liver bud. A key epithelial‐mesenchymal transition (EMT) event, primitive streak formation, is induced by Activin/Nodal signaling to convert the posterior epiblasts into blastic mesendoderms. Activation of Wnt signaling is also a crucial event in primitive streak formation from epiblasts prior to Activin/Nodal signaling.^11^, ^12^, ^13^, ^14^ The foregut endoderm is then also specified by a Wnt signal gradient within the primitive streak region. Hepatoblast specification occurs through another EMT event triggered by BMP2 and FGF4.^15^, ^16^, ^17^, ^18^ Therefore, efficient induction of two major EMT events is expected to increase HB‐LC induction from ESCs.^19^, ^20^, ^21^

First, we generated transgenic mouse ESCs carrying the CYP3A7R BAC reporter gene and investigated *CYP3A7R* expression in mice. Furthermore, we evaluated the possibility that *CYP3A7R* is reactivated during the process of liver regeneration in adult mice in response to liver injury. In addition, we tried to obtain basic information about the key factor(s) involved in the efficient in vitro production of HB‐LCs from mouse ESCs by measuring the fluorescence intensity or the number of CYP3A7R^+^ cells.

In this in vitro study, we focused on the processes occurring during the primitive streak and hepatoblast formation, and improved the previously reported method for differentiating HLCs from human iPSCs.^22^ We found that earlier Wnt signal activation and chromatin modulation increases the efficiency of HB‐LC formation. Moreover, we clearly captured the mitotic proliferation of RFP^+^ HB‐LCs initiated by BMP2 in real‐time, and treatment with 1.7% DMSO allowed selective HB‐LC proliferation. Finally, we confirmed that HB‐LCs could mature into HLCs by additional cell culture in a medium containing dexamethasone (DEX) and oncostatin M (OSM).^23^, ^24^ Using a microarray, immunostaining, and reverse transcriptase quantitative polymerase chain reaction (RT‐qPCR) analysis, we demonstrated that the HB‐LCs efficiently produced in this study were bipotent and capable of differentiating into HLCs.

## 2 EXPERIMENTAL METHODS

### 2.1 BAC vector construction

We previously created two knock‐in (KI) vectors, the LA‐DsRed‐pA‐RA‐CYP3A7 KI vector carrying a neomycin‐resistant gene and the loxP‐site KI vector,^9^ which we used to introduce a single CYP3A7R reporter gene into the RP11‐757A13 BAC clone (Invitrogen, Waltham, MA, USA). Recombinant DY380 colonies were then isolated as previously described.^9^

### 2.2 Cell culture

Details of all cell culture media used in this study can be found in Table S1. Mouse ESCs were cultured on mitomycin C‐treated mouse embryonic fibroblasts from E12.5 embryos in mouse ESC medium at 37°C with 5% CO_2_.

### 2.3 Generation of transgenic ESCs

An episomal mouse artificial chromosomal (MAC) vector was used as a stable carrier for the CYP3A7R BAC. MAC4 contains a loxP recombination site, a drug resistance gene, and an EGFP expression unit.^25^ We have also introduced a loxP site into the backbone of the CYP3A7R BAC vector. This allows one copy of the full length of CYP3A7R BAC to be loaded onto MAC4 through site‐specific recombination using the Cre‐loxP system. As a result, unexpected partial deletion is greatly reduced in both the BAC DNA and the genomic DNA of the integration site. In addition, MAC4 also contains a drug resistance gene adjacent to the loxP site, so periodic culture in a culture medium with a selection reagent can reduce the possibility that transgenes will be permanently silenced. Furthermore, MAC4 is stably maintained in mouse cells, and when transgenic mice are produced through chimeric mouse formation, all transgenic cells become positive for EGFP fluorescence, which makes it easy to identify them. The CYP3A7R MAC was transferred to female mouse TT2F ESCs *via* microcell‐mediated chromosome transfer.^26^ Transgenic ESC clones were selected by culturing them in ESC medium containing 800 µg/ml of G418. G418‐resistant clones were and characterized by genomic PCR and fluorescence in situ hybridization (FISH) mapping analysis. FISH mapping was performed following standard procedures.^9^ Subsequent experiments primarily used transgenic clone #5. Details of PCR primers used in this study can be found in Table S2.

### 2.4 Preparation of transgenic mice

Chimeric mice were generated using CYP3A7R transgenic ESCs. Briefly, ESCs were injected into eight‐cell stage embryos derived from ICR mice (CLEA, Tokyo, Japan) and embryos were then transferred to pseudopregnant ICR females. CYP3A7R transgenic mice were obtained by crossbreeding with 100% coat‐color chimeras. In this study, 40 chimera, 45 transgenic, and 16 nontransgenic control mice were analyzed.

### 2.5 Ethics statement

This study was carried out in strict accordance with the recommendations in the Institutional Animal Care and Use Committee of Tottori University. The Institutional Animal Care and Use Committee of Tottori University approved the protocol used in this study. The approval number for the generation of transgenic mice was h25‐049. Euthanasia was performed using the recommended method of manual cervical dislocation for small animals, including mice. Surgery was performed under anesthesia, and every effort was made to minimize suffering.

### 2.6 Genomic PCR and RT‐QPCR analysis

Genomic PCR was performed with 20 μl of TaKaRa Ex Taq mixture (TaKaRa Bio, Shiga, Japan). Primer sets used for genomic PCR analysis have been previously described.9 cDNA from 10 ng of RNA was amplified in 25 µl reactions using KOD SYBR® qPCR Master Mix (TOYOBO, Osaka, Japan) and a 7500 Real‐Time PCR System (Applied Biosystems, Waltham, MA, USA). *Actb* was used as an internal control. Details of primer sets used for RT‐qPCR can be found in the Table S2.

### 2.7 Induction of liver damage with CCl_4_

About 5% (v/v) carbon tetrachloride (CCl_4_) (Wako, Osaka, Japan) was diluted with corn oil (Sigma‐Aldrich, St. Louis, MO, USA) and 5 ml of CCl_4_ *per* kg of body weight was intraperitoneally administered to nine 8‐weeks‐old (8W) CYP3A7R (±) mice (n = 3). Three treated mice *per* day were dissected on days 1, 2, and 3 after treatment. Three untreated mice were used as a negative control (day 0).

### 2.8 Immunostaining

Paraffin sections were prepared from mouse tissues fixed with PBS containing 4% (w/v) paraformaldehyde (PFA) for 10 minutes. Cells cultured on dishes were fixed with 2% PFA for 5 minutes. The deparaffinized tissue sections and cultured cells were incubated with PBS containing 2% skim milk (Difco, San Diego, CA, USA) for 30 minutes at room temperature. Details of all antibodies used in this study can be found in the Table S3. The primary and secondary antibodies were diluted with PBS containing 2% skim milk at a dilution of 1:500. Nuclei were stained with Prolong Gold antifade reagent containing DAPI (Life Technologies, Waltham, MA, USA).

### 2.9 Hepatoblast induction from mouse ESCs

Embryoid bodies (EBs) were formed from mouse ESCs and cultured in 10% FBS medium for 1‐3 days in noncoated dishes to induce epiblast‐like cells (EpiLCs). EBs were placed in plates and incubated for 24 hours in 10% FBS medium supplemented with 5 µmol/L 5‐azacytidine (5azaC, Sigma‐Aldrich, St. Louis, MO, USA). The medium was replaced with 10% KSR medium. Then, 6 µmol/L CHIR99021 (Stemgen), 100 ng/mL of Noggin (R&D Systems, Minneapolis, MN, USA), and 50 nmol/L trichostatin A (TSA, Sigma‐Aldrich, St. Louis, MO, USA) were added for the first day. To induce primitive streak formation, 10 ng/mL of Activin A in 10% KSR medium was added for 1 day. Cells were then cultured in KSR medium containing 0.1% insulin transferrin selenium (ITS) (Invitrogen, Waltham, MA, USA) for 1 day, and then 1% ITS was added for 1 day. Next, the cells were cultured in 10% KSR medium containing 30 ng/ml of FGF4 (PeproTech, Cranbury, NJ, USA) and 15 ng/ml of BMP2 (PeproTech, Cranbury, NJ, USA) for 4 days. The hepatic cell population was then cultured in a hepatoblast growth medium (HB growth medium). To enrich HB‐LCs gradually, 0.2 to 0.4% DMSO (Sigma‐Aldrich, St. Louis, MO, USA) was added to the HB growth medium. At the end of the cell culturing period, 1.7% DMSO was added to the HB growth medium for two weeks. HB‐LCs prepared from wild‐type (WT) ESCs were further cultured in maturation medium.

### 2.10 Fluorescence microscopy and image analysis

Fluorescent images of CYP3A7R cells were captured using a BZ‐9000 fluorescence microscope (Keyence, Osaka, Japan), an A1 confocal microscope (Nikon, Tokyo, Japan), or a VivaView FL (LCV‐110) incubator fluorescence microscope (Olympus, Tokyo, Japan). Live cell nuclei were stained with medium containing 1 µg/ml of Hoechst 33 342. The average and standard deviation of the total area fluorescence and numbers of RFP^+^ cells were calculated using ImageJ and BZ‐9000 software.

### 2.11 Microarray expression analysis

GeneChip® (3'IVT) array expression analysis (Thermo Fisher Scientific, Waltham, MA, USA) was performed using RNA extracted from day‐41 cells differentiated from mouse ESCs, embryonic 18.5‐day (E18.5) fetal mouse liver (FL), and adult 6W mouse liver (AL). The expression profile was analyzed only for highly expressed genes with actual log_2_ values ≥ 10. Genes with more than a 2‐fold increase or decrease in log_2_ values compared to other samples were considered differentially expressed genes. Heatmaps were created using the free R‐family software RStudio (Version 1.2.5033).

### 2.12 Statistics

Data are presented as mean ± standard deviation. The Mann‐Whitney *U* test was used to evaluate differences between treated samples and controls. Significance was determined using equal‐variance Z values on both sides. Values of *P* < .01 were considered significant. *, *P* < .05; **, *P* < .01; ***, *P* < .001.

## 3 RESULTS

### 3.1 Generation of CYP3A7R transgenic ESCs and mice

**Figure 1:**
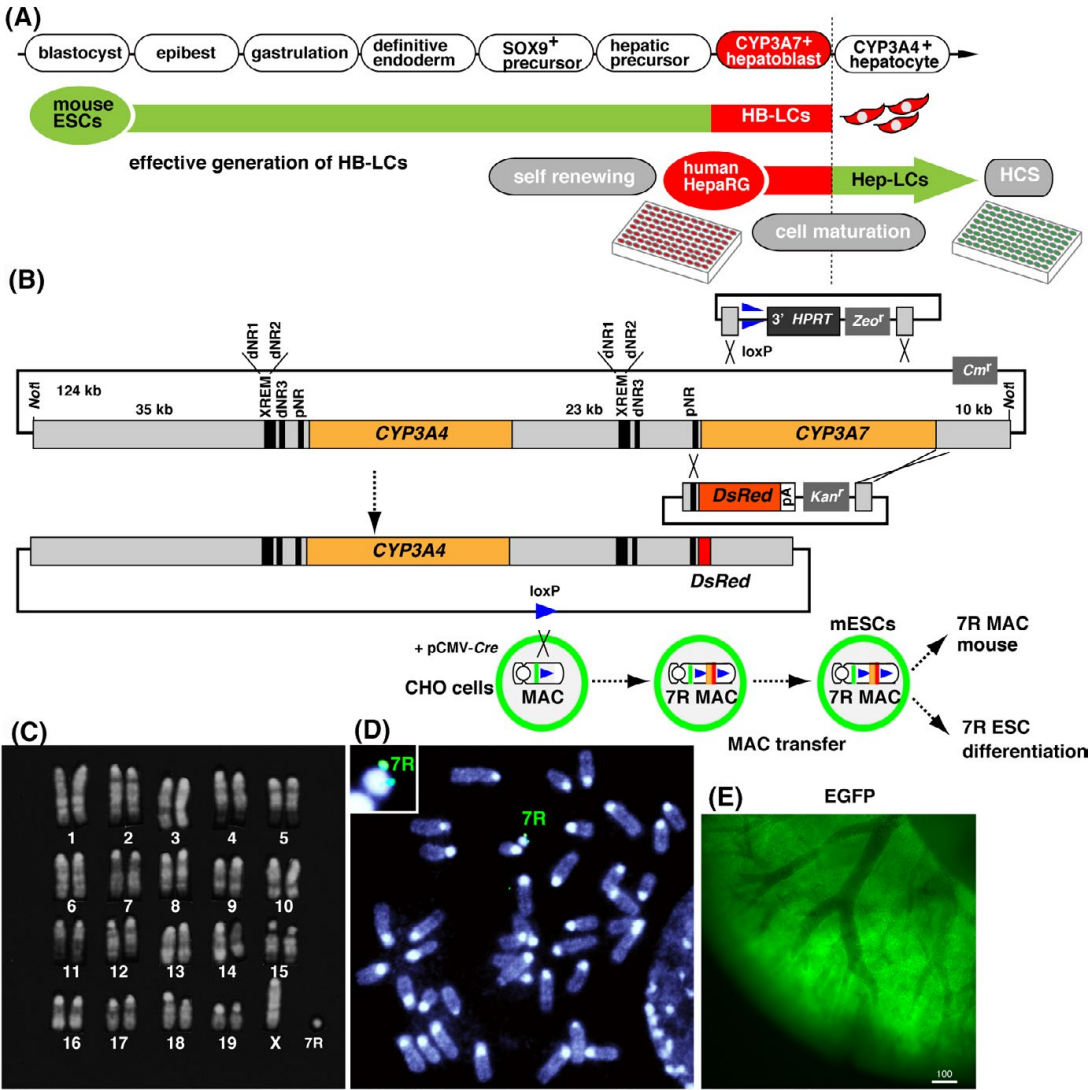
Recombineering of RP11‐757A13 BAC to monitor CYP3A7 expression with red fluorescence. A, An overview of the projected experimental design. B, Two knock‐in vectors (top) and the resulting CYP3A7R BAC (bottom). Orange, human *CYP3A4* ORF; red, *DsRed*; triangle, loxP; black vertical lines, transcriptional regulatory elements. C, Karyotype of transgenic mouse ESCs. D, FISH signal for CYP3A7R BAC (green) in the MAC. E, Ubiquitously expressed EGFP derived from a transgene in the MAC vector

Figure 1A presents an overview of the projected experimental design for the generation of homogeneously mature HLCs from PSCs. In this study, as an initial part of a long‐term plan, we aimed to find a procedure for the generation of HB‐LCs. Previously, the entire human *CYP3A* gene cluster was placed on a chromosomal vector, and then introduced into mice lacking the mouse *Cyp3a* gene cluster. In the humanized CYP3A mice, *CYP3A7* was specifically expressed in fetal liver and small intestine by 6 weeks after birth,^8^, ^27^ suggesting that the *CYP3A7* reporter would be possible to use as a general hepatoblasts‐specific marker in human and mice. To use the property of *CYP3A7*, the CYP3A7R BAC reporter gene was introduced into mouse ESCs using a chromosomal vector, MAC4. First, the entire region encoding *CYP3A7* was exchanged with *DsRed* cDNA on the RP11‐757A13 BAC clone, which contains the complete genomic regions of human *CYP3A4* and *CYP3A7* arranged in tandem (Figure 1B). The CYP3A7R BAC retains the whole region of *CYP3A4*, and so *CYP3A4* was expected to be expressed in mature hepatocytes of transgenic mice.

The MAC4 vector contained a ubiquitously expressed *EGFP* gene to facilitate the identification of transgenic ESCs and mice. The reconstructed MAC was introduced into mouse TT2F ESCs (2n = 39, XO), and transgenic CYP3A7R ESC clones were selected for G418 resistance, conferred by the *neo* gene in the knock‐in vector and green fluorescence. The karyotype of isolated clones was 2n = 40, XO, +MAC (Figure 1C). FISH mapping demonstrated that the CYP3A7R BAC was present in the MAC vector in transgenic mouse ESCs (Figure 1D). Next, we created transgenic mice through chimera formation using a CYP3A7R ESC clone. Transgenic mice strongly expressed EGFP (Figure 1E) ubiquitously, demonstrating that the episomal CYP3A7R MAC vector was stably maintained.

**Figure 2:**
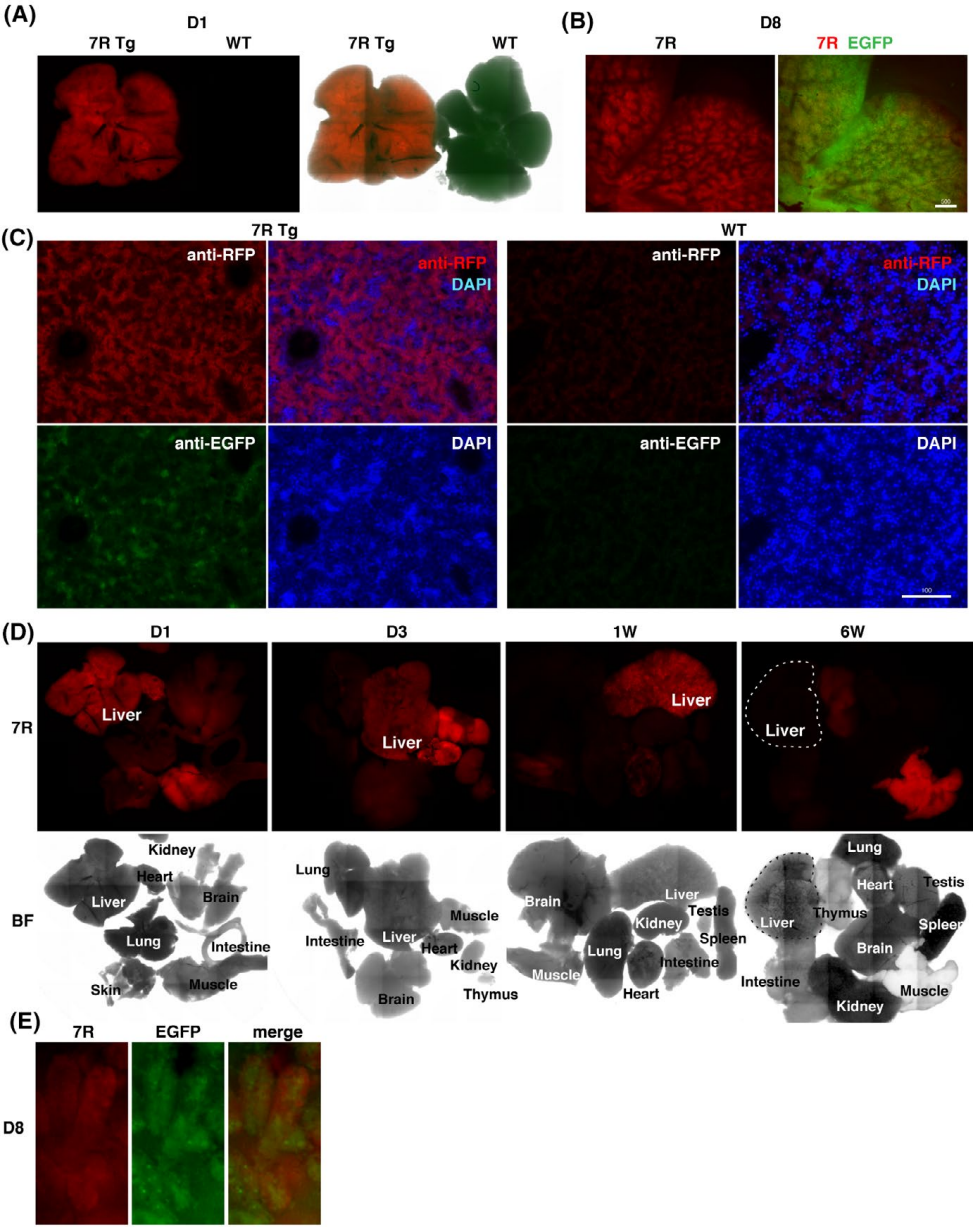
Parenchymal hepatocytes specifically express RFP in the newborn liver. A, 7R, CYP3A7R transgenic liver; WT, wild‐type liver on day 1, a negative control. B, Transgenic hepatic lobules on day 8. Scale bar, 500 µm. C, Liver sections immunostained for RFP on day 1. Scale bar, 100 µm. D, CYP3A7R mouse organs. 7R, RFP fluorescence signals; BF, bright field. E, Enlarged image of intestinal microvilli on day 1

CYP3A7R newborn pups strongly expressed RFP in the liver (Figure 2A), and red fluorescence was limited to the hepatic lobule (Figure 2B). Immunostaining with the anti‐RFP antibody on paraffin‐embedded sections demonstrated that RFP expression was limited to hepatic parenchymal cells in transgenic newborn mice (Figure 2C). Therefore, the CYP3A7R reporter is specifically expressed in premature hepatic parenchymal cells in the transgenic liver. We also analyzed the developmental regulation of CYP3A7R in transgenic embryos at E14.5 and E18.5, and postnatal mice at day 1 (D1) to 12W. The fluorescence intensity of RFP was strong 1 day after birth and decreased gradually each day after that. Most of the other organs in transgenic newborn mice were negative for RFP fluorescence. At approximately 6W after birth, red fluorescence was no longer visible (Figure 2D). We observed RFP in the outer layer of intestinal microvilli (Figure 2E), which expresses CYP3A7 in humans. Unexpectedly, skeletal muscles and cardiomyocytes in almost all CYP3A7R mice exhibited strong red fluorescence (Figure 2D), which could have been caused by either leaking of strong EGFP fluorescence into the red fluorescence channel or tissue‐specific autofluorescence. Thus, the mRNA expression of *CYP3A7R* was analyzed using RT‐qPCR.

**Figure 3:**
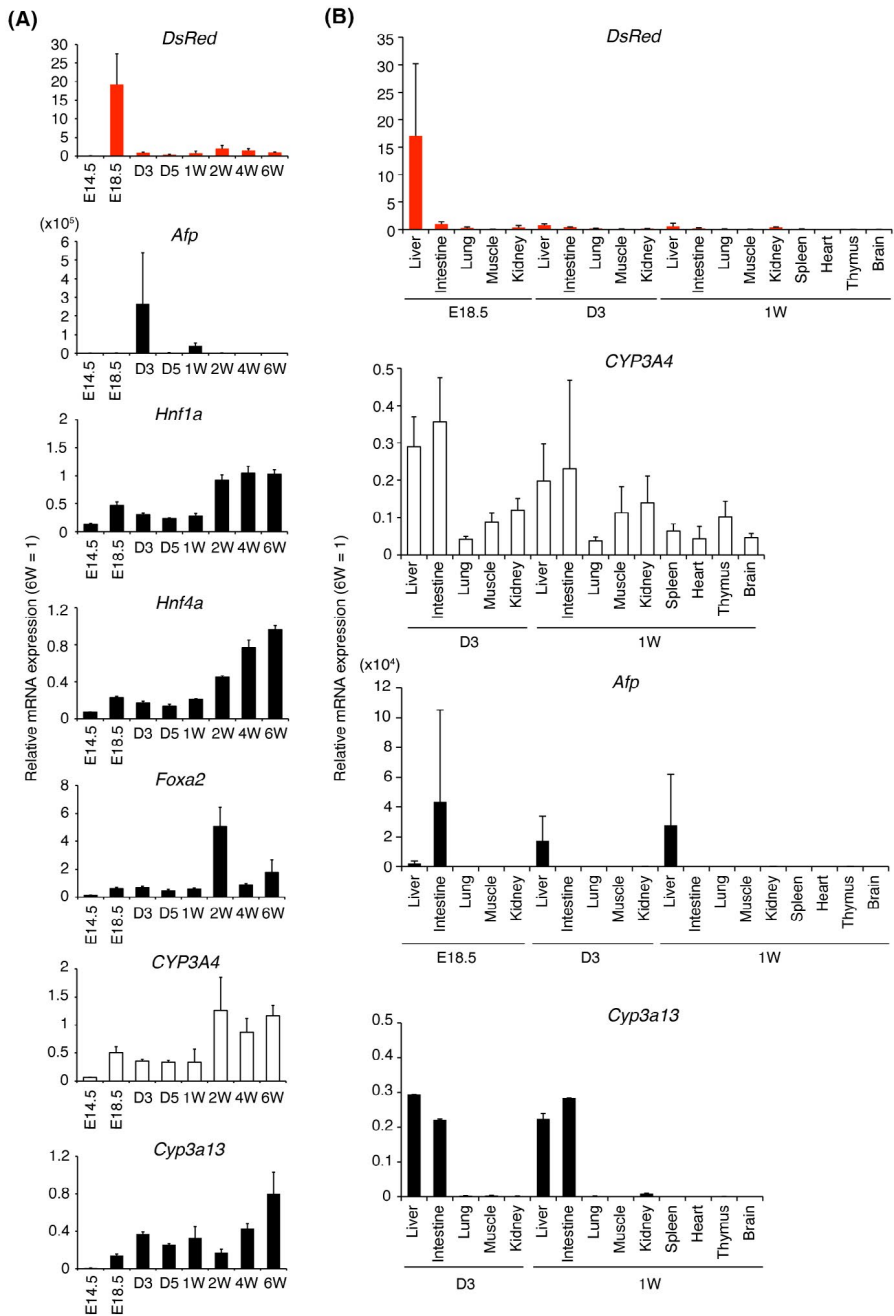
mRNA expression in CYP3A7R mice. A, RT‐qPCR analysis of *DsRed* and *Afp* in the perinatal liver and abundantly expressed genes in hepatocytes. Values are normalized to 6W transgenic liver expression levels. B, Tissue‐ and stage‐specific expression (n = 2, duplicate measurements)

We analyzed mRNA levels of *DsRed*, *Afp* (an endogenous marker for fetal liver), *CYP3A4*, and *Cyp3a13* (an ortholog of human *CYP3A* and shares 75% amino acid homology with CYP3A4.28 *Hnf1a*, *Hnf4a*, and *Foxa2* encode transcriptional factors involved in the formation of multiple endodermal organs, including liver and pancreas during embryonic development, and additionally function in differentiated tissues.^28^, ^29^, ^30^, ^31^ We then compared gene expression levels in livers and various tissues from CYP3A7R mice (n = 2, triplicate measurements) at E18.5; at postnatal days 3 and 5 (D3 and D5, respectively); and 1, 2, 4, and 6 weeks after birth. Interestingly, *DsRed* and *Afp* were transiently upregulated at E18.5 and D3, respectively. Thereafter, the expression of these fetal genes was silenced (Figure 3A). In contrast, expression of BAC‐derived exogenous *CYP3A4* mRNA increased slightly at E18.5 and then further after 2W. Similar expression profiles were seen for *CYP3A4* in *Hnf1a*, *Hnf4a*, and *Cyp3a13. Foxa2* was upregulated at 2W (Figure 3A). At E18.5, *DsRed* mRNA was specifically expressed in the liver and slightly in the intestine (Figure 3B). *Cyp3a13* was expressed in the liver and the intestine of D3 and 1W mice (Figure 3B). Neither *DsRed*, *Afp*, nor *Cyp3a13* was expressed in muscles and hearts. Therefore, the human transgene *CYP3A7R* is likely to be developmentally and tissue‐specifically regulated in mice. Furthermore, in our in‐vitro differentiation procedure, neither cardiac nor skeletal muscles were co‐induced together with HB‐LCs. Therefore, HB‐LCs can be specifically monitored using red fluorescence during in‐vitro hepatic cell differentiation.

### 3.2 | Reactivation of CYP3A7R in response to acute hepatic injury in adult MICE

**Figure 4:**
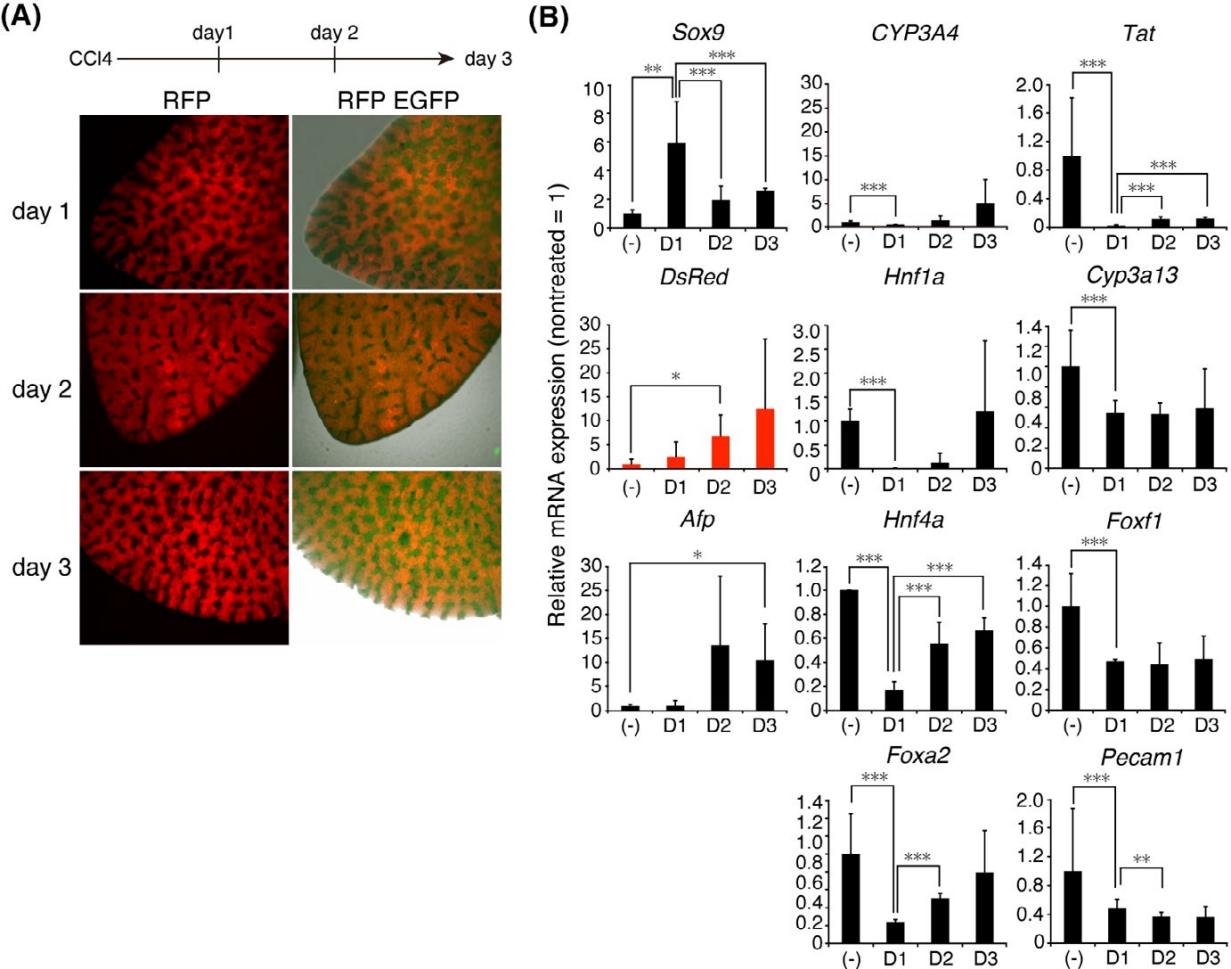
Reactivation of CYP3A7R after hepatic injury. A, Livers of adult CYP3A7R mice on days 1 to 3 after CCl_4_ treatment. B, RT‐qPCR analysis in untreated (‐) and treated livers (n = 3, triplicate measurements). Values for treated livers are normalized to those of untreated livers

We used our fluorescent reporter to visualize hepatic regeneration after injury in adult mice. After administration of 5% (v/v) CCl_4_ to induce liver damage, we observed that RFP increased each day for 3 days (Figure 4A). The RT‐qPCR analysis demonstrated that mRNA expression of *Sox9*, *Afp,* and *DsRed* was significantly upregulated on day 1, day 2, and day 3 after CCl_4_ treatment, respectively, in the affected liver (Figure 4B, n = 3, triplicate measurements). SOX9 is a transcriptional factor expressed in stem cells that differentiate into intestinal, hepatic, and pancreatic cells and also in epithelial cells.^32^ In contrast, mRNA expression of *CYP3A4*, *Hnf1a*, *Hnf4a, Foxa2*, and *Tat* was downregulated on day 1 but recovered on day 3. Expression of *Cyp3a13, Foxf1* (an endogenous marker of stellate cells), *and Pecam* (an endogenous marker of biliary epithelium) did not recover by day 3.^33^, ^34^ This result suggests that liver injury induced by hepatotoxic substances heals quickly due to rapid regeneration of hepatocytes and that the moderate hepatotoxicity of administered chemicals could be conveniently assessed in real‐time by measuring the increase in the level of RFP using transgenic HLCs.

### 3.3 Efficient induction of HB‐LCs from mouse CYP3A7R ESCs in vitro

**Figure 5:**
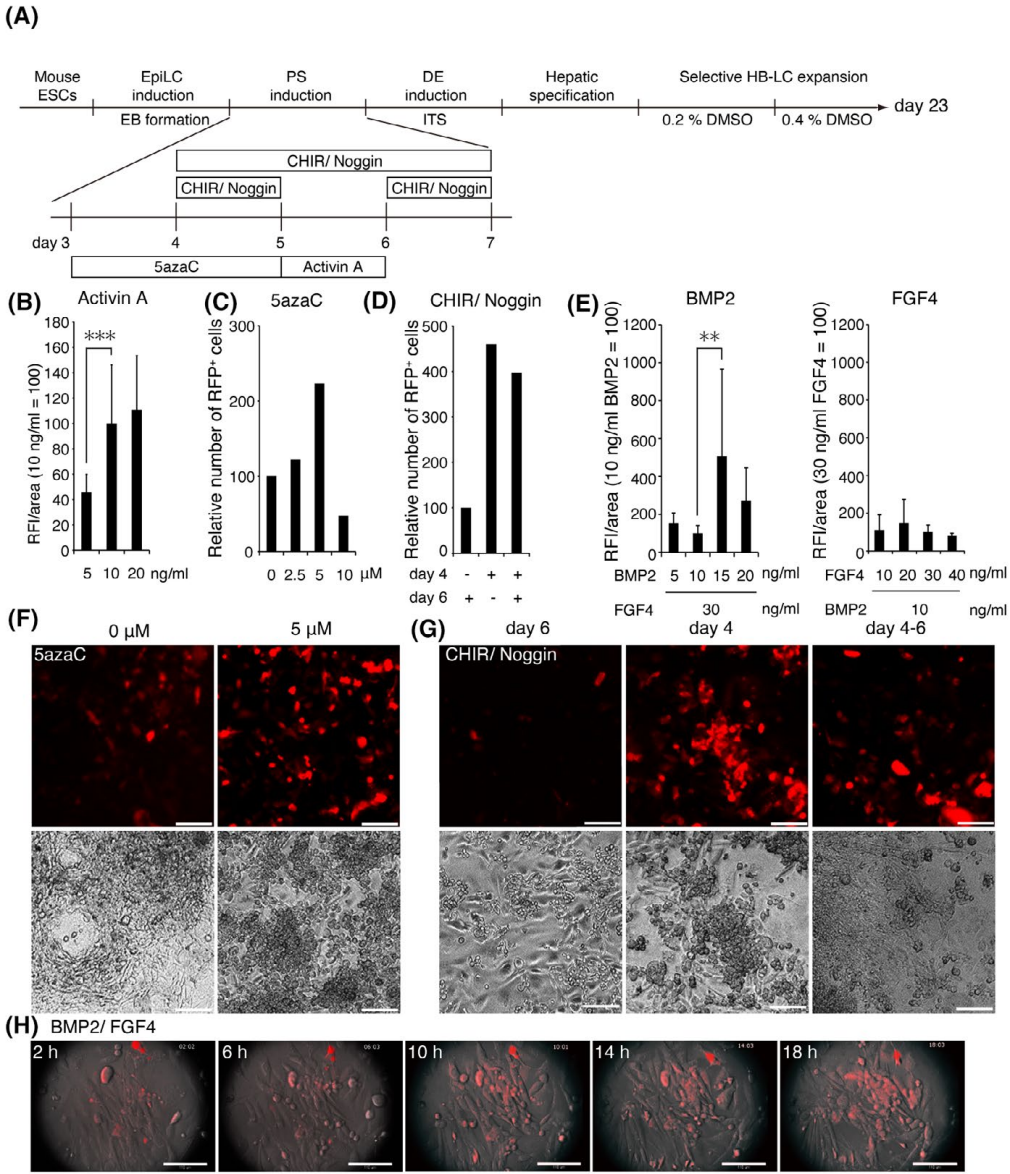
Semi‐quantitative fluorometric evaluation of HB‐LCs. A, A flow chart detailing the assay procedure (n = 2, triplicate measurements). PS, primitive streak; DE, definitive endoderm. B, Cells were incubated with 5‐20 ng/mL of Activin A for 24 h on day 5. (C, F) Cells were incubated with 0‐10 µmol/L 5azaC for 48 h on day 3. (D, G) Cells were incubated with 6 µmol/L CHIR99021 and 100 ng/mL of Noggin for 24 h on day 4, 24 h on day 6 or 72 h on day 4. Scale bar, 100 µm. E, Cells were incubated with 5‐20 ng/mL of BMP2 and 30 ng/mL of FGF4 (right) or with 10‐40 ng/mL of FGF4 and 10 ng/mL of BMP2 (left). H, Still images from an 18‐hour movie taken during incubation with BMP2/FGF4. Scale bar, 110 µm

Based on the results of the in vivo experiments, we modified a previously reported human HLC induction method^22^ to optimize each step of mouse HB‐LC differentiation based on levels of total red fluorescence intensity in a given area (RFI/area) and/or the relative number of RFP^+^ cells. The 23‐day cell culture period was subdivided into four stages: EpiLC formation, primitive streak formation, definitive endoderm induction, and hepatic specification (Figure 5A). We initially used one‐day‐old EBs but discontinued their use due to their poor adhesion to cell culture plates. Therefore, we often used three‐days‐old EBs, as they display good adhesion efficiency to the bottom of the culture plate. In a previous report, 100 ng/ml of Activin A was used to induce primitive streak formation in human iPSCs, but we found that 10 ng/ml of Activin A was sufficient in mouse cells (Figure 5B). In particular, we found that chromatin modulation was critical for the efficient induction of primitive streak formation. We treated EpiLCs with 5 µmol/L of the DNA demethylation agent 5azaC for 48 hours, which we expected to increase the number of definitive endoderm cells. Consequently, RFP^+^ cells were more likely to be induced during the differentiation of primitive endoderm into HB‐LCs (Figure 5C,F). The number of RFP^+^ cells in a population treated with CHIR99021 (glycogen synthase kinase‐3 inhibitor) and Noggin (BMP inhibitor) on day 4 prior to treatment with Activin A on day 5 was 4.6 times greater than in a population treated on day 6 (Figure 5D,G).

Next, hepatoblast‐specification factors (10 ng/mL of BMP2 and 30 ng/ml of FGF4) were added, which rapidly accelerated RFP^+^ cell proliferation (Figure 5E, H, and Movie S1). In particular, the concentration of BMP2 strongly affected the efficiency of HB‐LC induction; treatment with 15 ng/mL of BMP2 increased the number of RFP^+^ cells 5‐fold compared with treatment with 5 ng/mL of BMP2 (Figure 5E).

**Figure 6:**
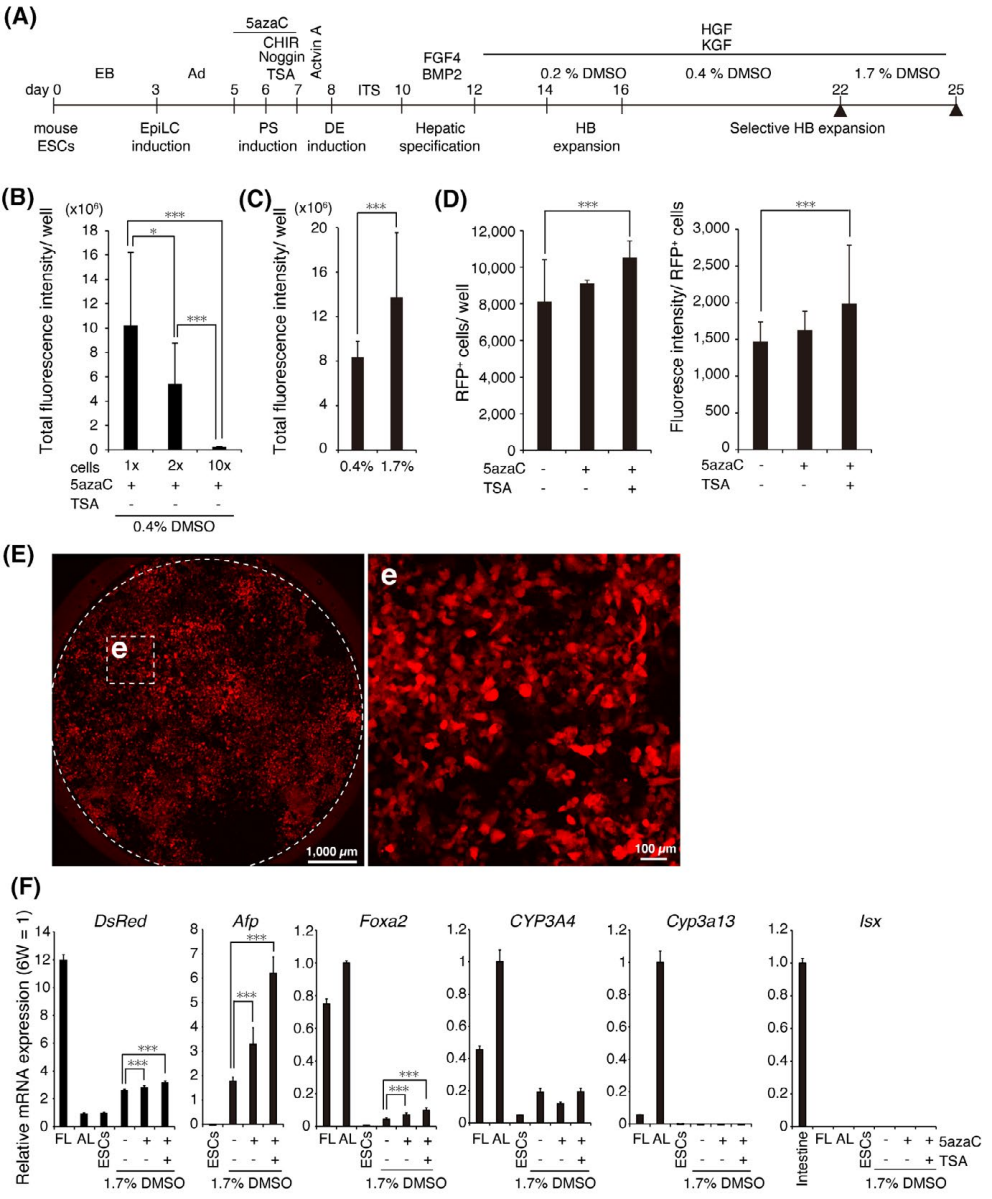
Efficient induction of HB‐LCs from mouse ESCs. A, Scheme of HB‐LC induction (n = 2, triplicate measurements). Ad, adhesion; PS, primitive streak; DE, definitive endoderm; HB, hepatoblast. B, Number of ESCs seeded into each well of a 96‐well plate. 1 ×, 7 × 10^5^ cells/well. Treatment with 1.7% DMSO (C), a combination of 5azaC and TSA (D) increases the number of RFP^+^ cells. (E) The day‐25 RFP^+^ cell image. Scale bar, 1000 µm (left). (e) Enlarged images of boxed areas in (E). Scale bar, 100 µm. F, Relative mRNA levels in day‐25 cells. The levels of most of the genes were normalized to those of the 6W adult liver. The expression levels of *Isx* were normalized to those of the 6W adult intestine. FL, E18.5 fetal liver; AL, 6W adult liver; ESCs, undifferentiated transgenic ESCs

The presence of DMSO at a high concentration matures hepatic precursors and also eliminates nonhepatic cells lacking metabolic activity, leading to enrichment of functional HLCs.^10^ We increased the concentration of DMSO in the culture medium in a stepwise fashion from 0.2% to 0.4% over 9 days and to 1.7% for the final 3 days (Figure 6A). First, we optimized the number of mouse ESCs to seed in each well of a 96‐well plate and found that EBs formed from 7 × 10^5^ ESCs produced many RFP^+^ HB‐LCs (Figure 6B). The addition of 1.7% DMSO further increased the total RFP fluorescence intensity/well (two wells, four independent areas *per* well) (Figure 6C). The total RFP fluorescence intensity/well was also increased by treatment with 5 µmol/L 5azaC (Figure 6D). We further investigated the effect of the histone deacetylase inhibitor TSA as another chromatin modulator, which increased the number of RFP^+^ cells/well and fluorescence intensity/cell (Figure 6D), suggesting that treatment with 5azaC and TSA had increased the number of HB‐LC precursors. Finally, we were able to prepare 96‐well plates full of RFP^+^ cells (Figure 6E).

Next, we briefly characterized the day‐25 cell population by comparing the level of *DsRed* mRNA with the level of E18.5 transgenic fetal mouse liver using RT‐qPCR (Figure 6F). The level of *DsRed* mRNA in the day‐25 cells was approximately 37% of that in the liver of E18.5 transgenic mice. The expression of *Afp* mRNA was also upregulated. In addition, the expression of *CYP3A4 and Foxa2* was also gradually upregulated. However, *Cyp3a13* (a major mouse adult‐type P450) and *Isx* mRNA (an endogenous marker of intestinal cells^35^) were undetectable. These results indicate that the RFP^+^ cells are proliferative HB‐LCs.

### 3.4 Maturation culturing of HB‐LCs to HLCs

**Figure 7:**
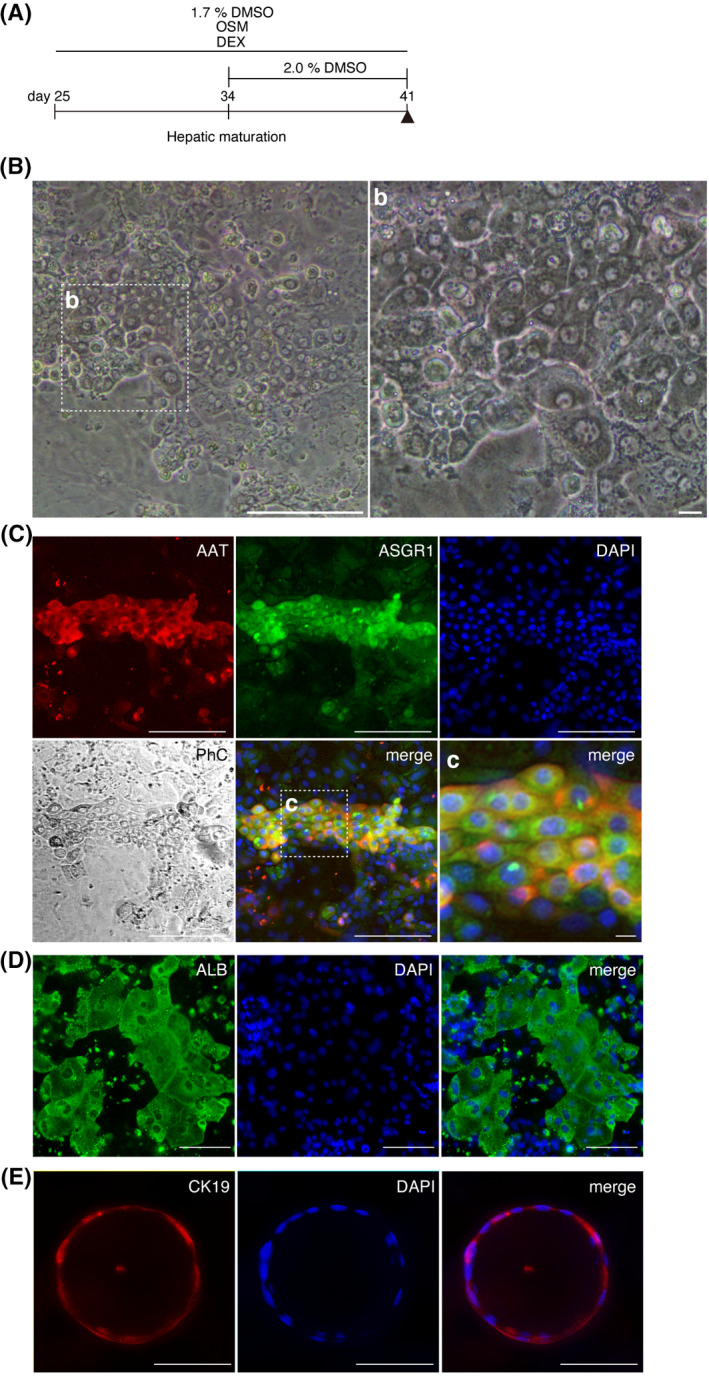
HB‐LCs capable of differentiating into HLCs and bile ducts. A, Maturation culture method (n = 4). The day‐41 cells matured from day‐25 HB‐LCs. B, Phase‐contrast microscope image of the day‐41 cells. Scale bar, 100 μm (left). (b) Enlarged image of the framed area in (B). Scale bar, 10 μm. C, Immunostaining for mature hepatocyte markers, AAT, and ASGR1. Scale bar, 100 μm. (c) Enlarged image of the framed area in (C). Scale bar, 10 μm. D, Immunostaining for ALB. E, Biliary epithelium marker, CK19. Scale bar, 100 μm

Next, we applied our HB‐LC induction method to WT mouse ESCs. We then extended the cell culture period to investigate whether HB‐LCs are bipotent and can mature into HLCs. Briefly, the day‐25 cells were cultured for 16 days in medium containing 1.7% DMSO, 100 nmol/L DEX, and 10 ng/ml of OSM. In some cases, cells were cultured in 2.0% DMSO medium for the last week (Figure 7A). Dinuclear cells became prominent in the day‐41 cells (Figure 7B). The day‐41 cells were positive for AAT and ASGR1 (Figure 7C). Some cells were positive for anti‐ALB antibody staining (Figure 7D) and others were positive for CK19 antibody, a cholangiocyte‐specific marker (Figure 7E). These results indicate that our culture method can efficiently generate bipotent HB‐LCs, which can eventually mature into HLCs.

### 3.5 High levels of expression of hepatocyte‐specific markers in HLCs

**Figure 8:**
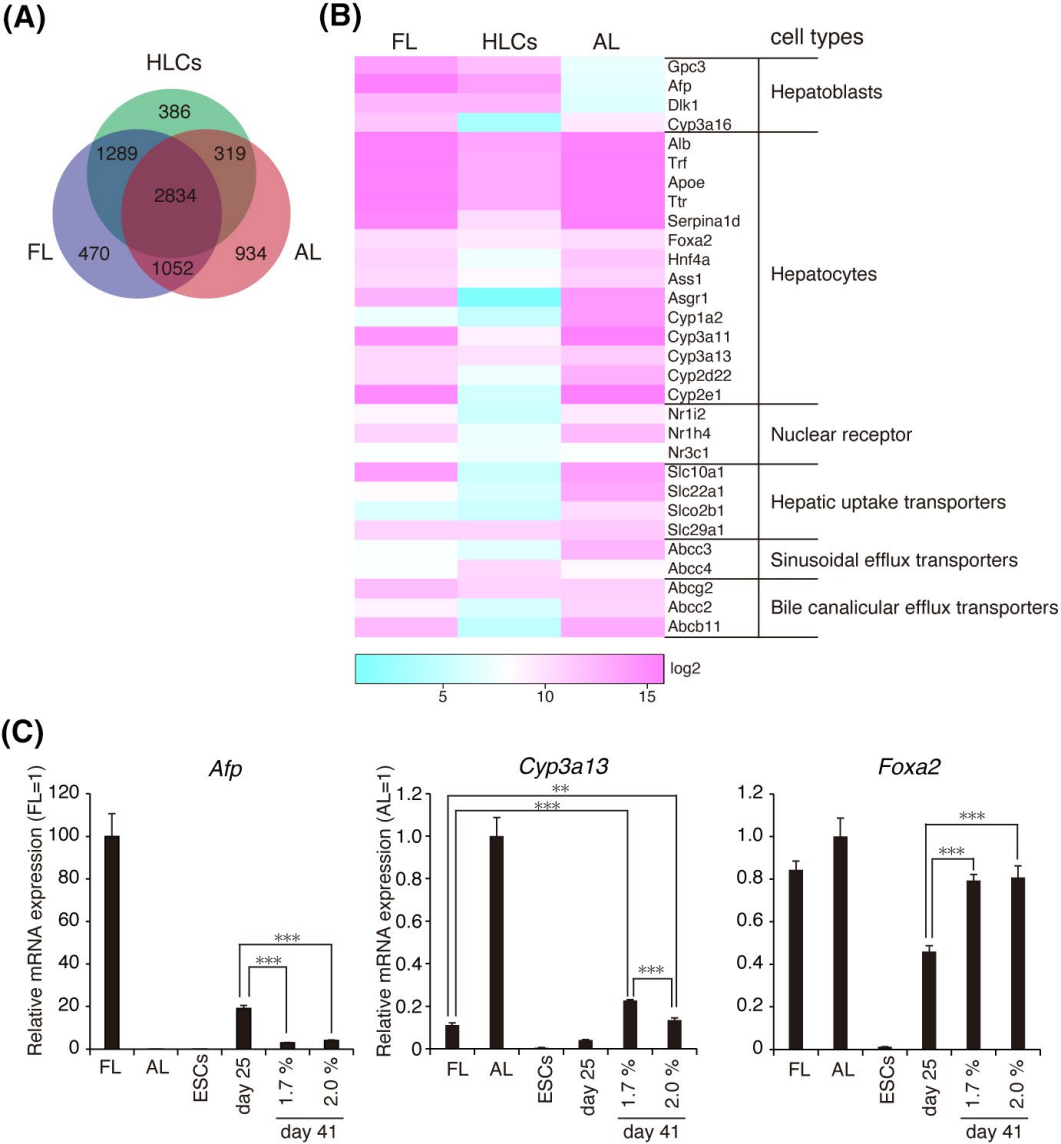
Cell maturation from HB‐LCs to HLCs. A, Venn diagram shows the number of differentially or similarly expressed genes detected among E18.5 FL, 6W AL, and day‐41 cells. B, Heat map showing the gene expression profile as log_2_ values for a subset of genes that were differentially and/or similarly expressed between them. We compared 30 genes expressed in the liver. Scale bar, log_2_ transformed normalized expression. C, Relative mRNA levels in the day‐41 cells. Values were normalized to 6W AL or E18.5 FL values

The GeneChip analysis was used to compare highly expressed genes in the day‐41 cells, E18.5 FL, and 6W AL. Approximately 65% and 48% of the highly expressed genes in day‐41 cells are similar to those in AL and FL, respectively, indicating that day‐41 cells contain a large number of HLCs. (Figure 8A). Using a heat map, we compared the expression levels of the representative genes for hepatoblasts, hepatocytes, nuclear receptors, hepatic uptake transporters, sinusoidal efflux transporters, and bile duct efflux transporters between samples (Figure 8B).^36^, ^37^, ^38^, ^39^ *Alb* was expressed at the AL level, while the hepatoblast‐specific genes were expressed at the FL level. In addition, CYP‐related genes and other marker genes were not sufficiently expressed in the day‐41 cells.

Finally, the mRNA expression profile was compared using RT‐qPCR between day‐25 cells and day‐41 cells (Figure 8C). The *Cyp3a13* gene was expressed at a level of approximately 22% in adult liver, whereas *Afp* mRNA expression was reduced throughout maturation cultures. In addition, another hepatocyte‐specific gene, *Foxa2*, was also upregulated. However, when the effects of 1.7% and 2.0% DMSO were compared, 2.0% DMSO was associated with lower levels of *Cyp3a13* expression, because high concentrations of DMSO are harmful and may affect the cell viability of early hepatocytes. These results suggest that the method presented here can be used for the efficient production of bipotential HB‐LCs.

## 4 DISCUSSION

We developed a CYP3A7R reporter system that can be used to follow cells expressing *CYP3A7* cording for fetal metabolic enzyme P450, which is specifically expressed in hepatoblasts. Red fluorescence produced by *CYP3A7R* functions as a sensitive label for hepatoblasts in transgenic mice and HB‐LCs. Red fluorescence is not observed in mature transgenic hepatocytes. Therefore, it is possible using this system to easily analyze the differentiation, proliferation, and maturation of hepatoblasts in vivo and in vitro. Additionally, *CYP3A7R* is temporarily reactivated in response to hepatic injury, demonstrating that HLCs derived from CYP3A7R PSCs can be used to establish an HCS system for the identification of hepatotoxic substances.

In this study, we focused on an EMT phenomenon initiated from posterior epiblasts during primitive streak formation. The resultant mesenchymal cells are the origin of the definitive endoderm. Signaling by Activin/Nodal, BMP, and Wnt/Ca2^+^ is involved in this event.^11^, ^12^, ^13^, ^15^, ^16^, ^17^, ^18^, ^19^, ^20^, ^21^ Subsequently, because BMP signaling negatively regulates definitive endoderm formation,^12^, ^13^ inhibition of BMP signaling by Noggin is required to enforce Wnt signaling at the anterior region of the primitive streak to form the definitive endoderm. Then, activation of BMP signaling by BMP2 is required to induce hepatoblasts from the definitive endoderm through another EMT event. Thus, complicated serial on/off switching of Wnt signaling and BMP signaling is theoretically required. However, we found that enforced activation of Wnt signaling preceding Activin/Nodal signaling leads to efficient induction of definitive endoderm (Figure S1).

Treatment with 1.7% DMSO matured hepatic precursors to HB‐LCs and HB‐LCs to HLCs. However, CYP‐related genes were not fully activated. Curiously, *Asgr1* mRNA expression was not detected by GeneChip analysis, but ASGR1 protein was detected by immunostaining in many binucleated cells. This discrepancy demonstrates that most of the cells were immature, even though some of them were already fully mature. Therefore, we conclude that the method presented here produces a uniformly differentiated HB‐LC population that can mature into HLCs and that HB‐LCs can be utilized for further optimization of the maturation culture method.

In the liver, hepatocytes are regularly renewed to maintain homeostatic liver function. New hepatocytes are supplied either through dedifferentiation of mature hepatocytes or maturation of Sox9‐expressing liver progenitor cells in response to liver injury.^40^, ^41^, ^42^, ^43^ SOX9^+^ liver progenitor cells retain their tissue‐of‐origin commitment and can differentiate into hepatic, pancreatic, and intestinal cells.^44^, ^45^ SOX9^+^ stem cells isolated from bile duct epithelium can form 3D organoids that mimic the liver and pancreas in vitro.^46^

In this study, expression of *Sox9* mRNA increased rapidly one day after CCl_4_ treatment and was followed by transcriptional induction of *DsRed* and *Afp*. Therefore, liver progenitor cells can be differentiated into HLCs *via* a transient cell state similar to that of HB‐LCs. Recently, it was reported that a combination of three small molecules termed YAC, consisting of Y‐27632 (Rho‐associated kinase inhibitor), A‐83‐01 (type transforming growth factor‐β receptor inhibitor) and CHIR99021, induces hepatic regeneration from mature hepatocytes.^47^ Such chemical induction of in situ hepatic regeneration may help recover compromised liver function and serve as an alternative method of regenerative medicine. When we added YAC to an HB growth medium, the mixture was cytotoxic for HB‐LCs. Therefore, reactivation of CYP3A7R may provide a semi‐quantitative evaluation system to identify reagents that initiate hepatic regeneration.

Previously, we produced transgenic HepaRG cells carrying a CYP3A4G/7R BAC reporter. The CYP3A4G/7R reporter is a dual‐color monitoring system in which HB‐LCs are labeled with RFP and CYP3A4‐expressing mature HLCs with EGFP.^9^, ^10^ It is worth noting that EGFP^+^ HepaRG‐derived HLCs can revert to RFP^+^ HB‐LCs. Thus, the CYP3A4G/7R reporter will be useful in developing a system that monitors both hepatic maturation and regeneration. It would be beneficial to perform preclinical assays under more physiological conditions using human PSC‐derived HLCs. Human iPSCs carrying the CYP3A4G/7R BAC reporter will be a useful tool in establishing an optimized method for producing human HLCs in the quantities required for regenerative medicine and drug discovery and development.

## DATA SHARING

The data discussed in this publication have been deposited in NCBI's Gene Expression Omnibus and are accessible through GEO Series accession number GSE152076 (https://www.ncbi.nlm.nih.gov/geo/query/acc.cgi?acc=GSE152076). Further information and requests for data and reagents should be requested to the corresponding author, Masako Tada. Please contact masako.tada@sci.toho‐u.ac.jp.

## DISCLOSURE

None of the authors has any conflicts of interest to declare.

## AUTHOR CONTRIBUTIONS

Masako Tada: research design, corresponding author, final proofreading before submission, collecting data, contributing to data analysis. Shota Okuyama, Fumihiko Kawamura, Musashi Kubiura, Saori Tsuji, Mitsuhiko Osaki, Yasuhiro Kazuki: conducted experiments, performed data analysis. Shota Okuyama: contributed to the writing of the manuscript. Hiroyuki Kugoh, and Mitsuo Oshimura: contributed new experimental tools. All authors reviewed the manuscript.

## ETHICAL STATEMENT

The authors declare that this study was performed under the research policy of Toho University and Tottori University. Details were described in the Experimental Methods section.

### OPEN RESEARCH BADGES

This article has earned an Open Data, Open Materials and Preregistration badges for making publicly available the digitally‐shareable data necessary to reproduce the reported results. The data is available in article supplementary material and also available on request from the authors. The GeneChip data will be displayed on the Gene Expression Omnibus (GEO) records. The permanent path to the registration is available at https://www.ncbi.nlm.nih.gov/geo/query/acc.cgi?acc=GSE152076.

## Supporting information

Fig S1

Table S1‐S3

Movie S1
