# Supplementary figures and images for: Real‐time fluorometric evaluation of hepatoblast proliferation in vivo and in vitro using the expression of CYP3A7 coding for human fetus‐specific P450
Source: Pharmacol Res Perspect. 2020 Sep 4;8(5):e00642. doi: 10.1002/prp2.642 (PMC7507068; doi:10.1002/prp2.642)

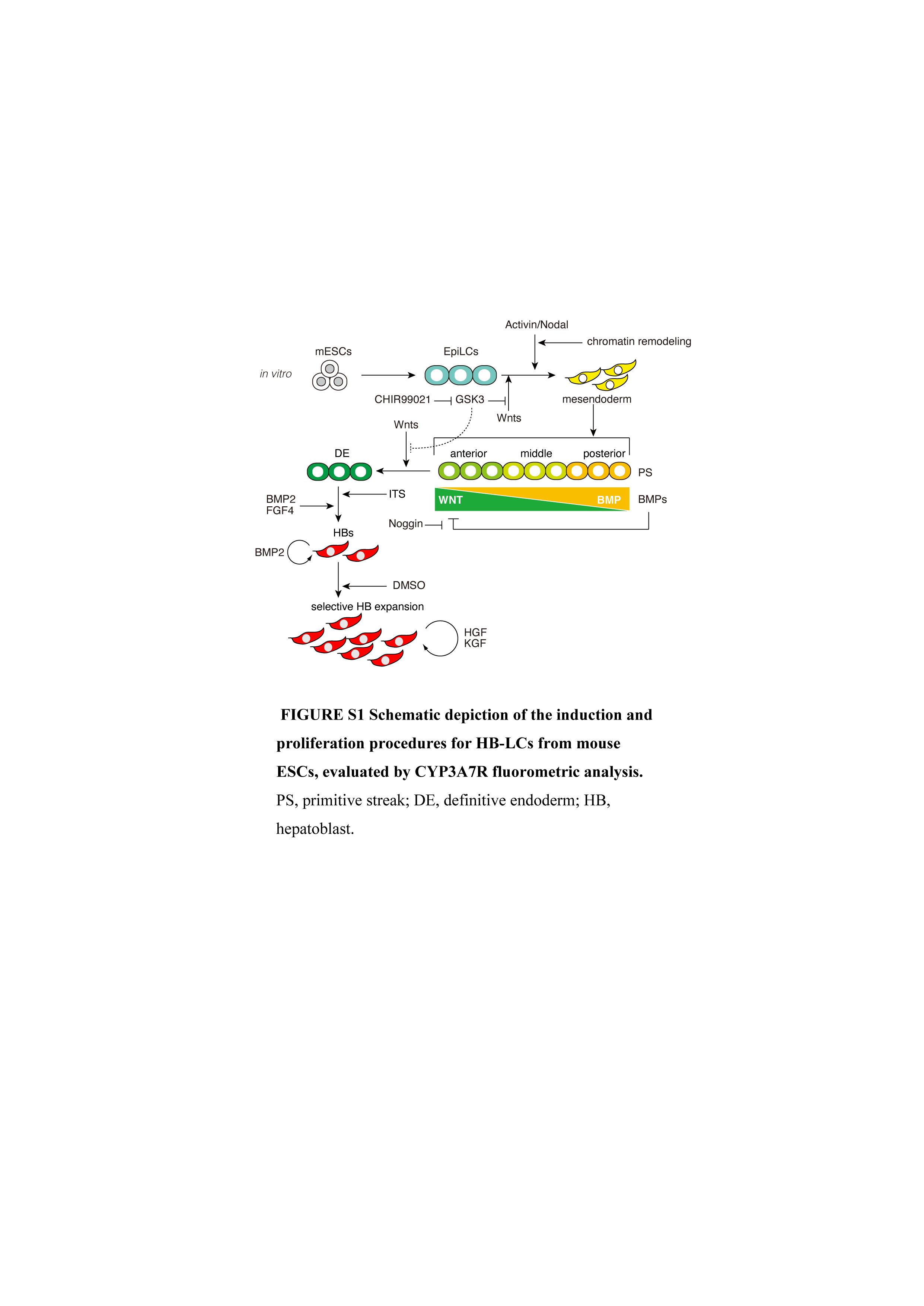

Supplement: Supplementary file 1 — Fig S1 [file PRP2-8-e00642-s001.tif]
