# Supplementary material for: Real‐time fluorometric evaluation of hepatoblast proliferation in vivo and in vitro using the expression of CYP3A7 coding for human fetus‐specific P450
Source: Pharmacol Res Perspect. 2020 Sep 4;8(5):e00642. doi: 10.1002/prp2.642 (PMC7507068; doi:10.1002/prp2.642)
Supplement: Supplementary file 2 — Table S1‐S3 [file PRP2-8-e00642-s002.docx]

Medium Contents

[D-MEM/Ham’s F-12 with L-glutamine (Wako) supplemented with

1 mouse ESC medium

10% fetal bovine serum (FBS, Corning), 1× penicillin-streptomycin

(P/S, Gibco®), 0.1 mM β-mercaptoethanol (2ME, Sigma-Aldrich) and 103 U/ml ESGRO® LIF (Merck Millipore)]

1. 10% FBS medium High Glucose D-MEM (Wako) with 10% FBS and 1×P/S

High Glucose D-MEM with 10% KnockOut™ Serum Replacement

1. 10% KSR medium
2. HB growth medium
3. maturation medium

(KSR, Gibco), 2 mM L-glutamine (Gibco), 0.1 mM 2ME and 10% nonessential amino acids

D-MEM with 10% FBS, 1× P/S, 1% sodium pyruvate, 10 mM nicotinamide, 2 mM L-ascorbic acid, 1% ITS, 10 ng/ml human HGF (Peprotech) and 20 ng/ml KGF (Peprotech)

710 HepaRG growth medium (Biopredic International) with 100 nM DEX (Sigma-Aldrich), 10 ng/ml OSM (Wako), and 1.7% DMSO

|  | Set | Gene | product | Primer name | Sequence (5' to 3') |
| --- | --- | --- | --- | --- | --- |
|  | 1 | *DsRed* | 67 | DsRed q-RT-F | GAAGGGCGAGATCCACAAG |
|  |  |  |  | DsRed q-RT-R | GGACTTGAACTCCACCAGGTA |
|  | 2 | *Afp* | 300 | AFP-F | CACTGCTGCAACTCTTCGTA |
|  |  |  |  | AFP-R | CTTTGGACCCTCTTCTGTGA |
|  | 3 | *CYP3A4* | 86 | hqCYP3A4-3A7-F | TTCATCCAATGGACTGCATAAAT |
|  |  |  |  | hqCYP3A4-R | TCCCAAGTATAACACTCTACACAGACAA |
|  | 4 | *Cyp3a13* | 59 | Cyp3a13-F | ACCGGCGGCGCTTT |
|  |  |  |  | Cyp3a13-R | ATTCTCAGAGATAGAGATGGCCTTTT |
|  | 5 | *Actb* | 115 | h/m Actb-F | CTTCTACAATGAGCTGCGTG |
|  |  |  |  | h/m Actb-R | GAAGGTCTCAAACATGATCTGG |
|  | 6 | *Hnf4a* | 62 | Hnf4a-F | CCAAGAGGTCCATGGTGTTT |
|  |  |  |  | Hnf4a-R | CCGAGGGACGATGTAGTCAT |
|  | 7 | *Foxa2* | 151 | Foxa2-F | GTGTACCGGACCAGGAGAAA |
|  |  |  |  | Foxa2-R | TTTGTGGAATTCTGGCCATT |
|  | 8 | *Hnf1a* | 108 | Hnf1a-F | TTACACCAAGTATCCCCCACA |
|  |  |  |  | Hnf1a-R | CAAGCTGTGCAGTGCTGTC |
|  | 9 | *Sox9* | 94 | Sox9-F | GTACCCGCATCTGCACAAC |
|  |  |  |  | Sox9-R | CTCCTCCACGAAGGGTCTCT |
|  | 10 | *Foxf1* | 95 | Foxf1-F | AGCATCTCCACGCACTCC |
|  |  |  |  | Foxf1-R | TGTGAGTGATACCGAGGGATG |
|  | 11 | *Pecam1* | 74 | Pecam1-F | CGGTGTTCAGCGAGATCC |
|  |  |  |  | Pecam1-R | ACTCGACAGGATGGAAATCAC |
|  | 12 | *Tat* | 84 | Tat-F | GTTGTCTGCCATTCCTGGAC |
|  |  |  |  | Tat-R | GGGAAGTGCTCCATCTCAAT |
|  | 13 | *Isx* | 75 | Isx-F | CCTGCCCTCAAACATGGA |
|  |  |  |  | Isx-R | TCTGTGGGAGGTACCAATGTAGT |
|  |  |  |  |  |  |

|  | antibody | host and conjugate | vendor |
| --- | --- | --- | --- |
| 1 | anti-DsRed | mouse monoclonal | Clontech |
| 2 | anti-EGFP | mouse monoclonal | TOYOBO |
| 3 | anti-AAT | rabbit polyclonal | DakoCytomation |
| 4 | anti-ASGR1 | mouse monoclonal | TOYOBO |
| 5 | anti-ALB | goat monoclonal | Bethyl |
| 6 | anti-CK19 | mouse monoclonal | DakoCytomation |
| 7 | anti-mouse IgG | Alexa Fluor® 546 goat monoclonal | ThermoFisher |
| 8 | anti-rat IgG | Alexa Fluor® 546 goat monoclonal | ThermoFisher |
| 9 | anti-rabbit IgG | Alexa Fluor® 488 goat polyclonal | ThermoFisher |
